# An Inexpensive Paper-Based Aluminum-Air Battery

**DOI:** 10.3390/mi8070222

**Published:** 2017-07-17

**Authors:** Ani Avoundjian, Vicente Galvan, Frank A. Gomez

**Affiliations:** Department of Chemistry and Biochemistry, California State University, Los Angeles, 5151 State University Drive, Los Angeles, CA 90032-8202, USA; aavound4@calstatela.edu (A.A.); vicentegalvan5@yahoo.com (V.G.)

**Keywords:** alkaline battery, aluminum, fuel cell, light-emitting diode, open circuit voltage

## Abstract

Paper-based batteries are an alternative to traditional batteries due to their low cost, portability, and simplicity to operate. In the present work, we demonstrate an improved and inexpensive paper-based aluminum-air battery employing KOH as the electrolyte with sufficient energy to power small devices. The dimensions of the device, electrode size, and electrolyte concentration were optimized with respect to amperage and reproducibility. The maximum amperage of 17.4 mA and maximum power of 3.0 mW was achieved with a 9 cm^2^ battery with anode and cathode electrode areas of 5.1 cm^2^ and 3.75 cm^2^ respectively, using 1.5 M potassium hydroxide (KOH). In a series configuration, the batteries generate sufficient energy to power light-emitting diodes (LEDs), a flashlight, a glucometer, and a pregnancy test.

## 1. Introduction

Alternative sources of power have become essential due to increasing global energy demand. Human-induced climate change drives the search for alternative and renewable energy sources, including solar energy, fuel cells (FCs), supercapacitors, and novel material batteries. Batteries are used in applications such as automobiles, cellular devices, computers, and other portable electronic devices, and are thus important energy sources to improve upon [1]. Aluminum was first introduced as a viable electrode for batteries in the 1850s by Hulot, when it was used as a cathode material in a zinc battery [2]. Several years later, aluminum was first used as the anode material in the Buff cell [3]. In the 1960s, Zaromb and Trevethan et al. introduced an aluminum/oxygen battery system; however, because of the formation of a non-reactive oxide layer on the aluminum under these conditions, the development of these types of batteries was not initially successful [4,5]. Thus, extensive efforts were made to develop a method of overcoming the formation of this oxide layer. One of the most effective methods was the use of aluminum alloys and doping the electrolyte with other compounds [6,7,8]. Among the metal/air batteries, aluminum/air batteries have a remarkable energy density (8.1 kW/kg) and a theoretical voltage of 2.71 V [9]. The low cost and ubiquitous nature of aluminum also makes it appealing for use as an anode for a variety of devices.

Traditional microfluidic devices (MDs) are composed of various polymers, most notably poly(dimethylsiloxane) (PDMS), which require external pumps to maintain fluid flow. Other substrates have been explored in an effort to reduce the cost of MDs, with one of the most prevalent being paper. Recently, paper material has been integrated into electrochemical devices due to its advantages over other platforms. Paper is ubiquitous, universally available, and an inexpensive alternative to other materials including glass and silicon [10]. Also, cellulose fibers make up the majority of various types of paper and allow the passive flow of fluids through capillary action, which consequently removes the need for external pumps [11]. Paper-like energy storage devices are becoming more attractive due to their easy integration with electronic devices [12]. Moreover, paper’s porosity makes it beneficial for the manipulation of electrons and ion transport throughout the structure [13]. Different types of paper can be used to manipulate flow rates and pore sizes depending on the desired output. There have been a myriad of paper electronic devices to date, which include electrochemical batteries, FCs, lithium-ion batteries, supercapacitors, and nanogenerators [10]. Despite these devices being more simplistic than conventional electronic devices, many still require complex fabrication. Paper has its disadvantages as well, which include its fragility. Paper may be subject to folding and tearing and is vulnerable in environmental settings unless encased in a protective layer. The first paper-based aluminum battery was demonstrated by Ferreria et al. using a layering structure of aluminum/paper/copper through thermal evaporation of the metal layers [14]. Using this structure, a current of 150 nA–0.5 mA was achieved. In this paper, different electrode materials and platform design were used, resulting in significantly less amperage and voltage compared to the current work. In 2013, Thom et al. as well as Zhang et al., showed improvement in the performance and feasibility of fabrication of a paper-based aluminum battery producing 1.3 V and 2.2 mA, and 1.53 V and 4.4 m/cm^2^, respectively [15,16]. In the work of Zhang, the batteries were used to power a glucose assay, demonstrating the potential application of this technology. These include providing sustainable power for point-of-care (POC) diagnostic devices, and other one-time use devices that currently employ button-cell batteries. There is a need to develop alternative power sources to button cell batteries, because they often times contain more energy than required and are environmentally hazardous when disposed of. The materials used in this aluminum-air battery can be reused either in the battery itself or in industrial processes. For example, steel mesh, activated carbon, and Kim Wipes, which are components of our aluminum-air battery, can be reused in the same battery after adding potassium hydroxide (KOH). The product of the anode reaction, aluminum hydroxide, Al(OH)_3_, is also a principal component of bauxite, which is used to manufacture alumina in the Bayer process. The resulting alumina is then used for the production of aluminum metal [17]. In this manuscript, we demonstrate a facile, cost-effective, and recyclable method of fabricating a paper-based aluminum-air flow battery with four fabrication steps, while generating power that is on par with other paper-based aluminum-air batteries (1.27 V and 3.4 mA/cm^2^) [15,16]. In addition, we also report on the optimization of the composition of the device, such as the cathodic material, the dimensions of the platform and electrodes, and the potential use in small applications. There was no specific algorithm used in the optimization; however, given that the voltage was relatively constant, optimization was mostly focused on maximizing the current as well as having a low relative standard deviation (RSD) of the amperage. When connecting the devices in series to ultimately power small devices, it was important to have not only high power, but also have high reproducibility in order for the devices to avoid being significantly affected by the weakest link.

## 2. Materials and Methods

### 2.1. Device Fabrication

The devices were fabricated using Kim Wipes (Fisher Scientific, Hampton, NH, USA), which were folded multiple times relative to their respective sizes ranging from 1–9 cm^2^. Kim Wipes were chosen as the paper substrate because of the faster flow rate of the electrolyte compared to chromatography paper. The anode was made from commercially available aluminum (Reynold’s Wrap, Reynolds Kitchens, Richmond, VA, USA), and was folded and placed directly onto the paper platform ranging from 0.75–6 cm^2^. The cathode, composed of various electrodes (Table 1), was added onto the platform and a current collector was folded over it to ensure even contact throughout the electrode. The activated carbon electrode was made of an ink consisting of an appropriate amount of black tempera paint (Michaels, Irving, TX, USA) and carbon to yield a percentage of 5% carbon to black paint [18]. Once the activated carbon was added to the black paint, the slurry was mixed by hand to ensure the even dispersion of carbon. The mixture was then painted on with a paintbrush. The silver electrode was composed of a silver conductive epoxy (MG Chemicals, Surrey, BC, Canada). The silver epoxy was similarly painted onto the battery. The epoxy consisted of two parts, A and B, which were mixed in equal amounts prior to painting onto the battery. Sodium hydroxide and potassium hydroxide (0.5–2.5 M) were used as the electrolytes, with a consistent electrolyte loading of 1 mL for each device. For each variation in the fabrication process, the battery samples were tested in triplicate.

### 2.2. Instrumentation and Testing Procedures

Experiments were carried out using a Princeton Applied Research 263A Potentiostat/Galvanostat with a two-electrode configuration (anode and cathode). Two different types of tests were performed on each individual battery: (i) an open circuit voltage (OCV) test of the battery was performed to determine the duration of the chip in terms of how long it maintains its voltage; (ii) voltage-current (VI) tests during which the current and voltage was recorded in 5 min intervals for 15 min. For the OCV test, the current was set to zero and the battery was tested after saturation. A linear sweep or VI test was also run, and the parameters were set to include a scan rate of 50 mV/s and a scan range from the OCV (i.e., the maximum potential) to 0.00 V (i.e., the minimum potential). In order to analyze the results, the potentiostat/galvanostat data for current and potential values was exported to a Microsoft Excel spreadsheet and plotted to create each tests’ corresponding polarization curve. The last test at 15 min was used to record the results for each battery recorded as an average value. From these results, polarization curves were produced, showing the maximum voltage, current, and power for an individual battery. Batteries were run in triplicate for each set of parameters that were changed.

## 3. Results

### 3.1. Working Principle

**Figure 1:**
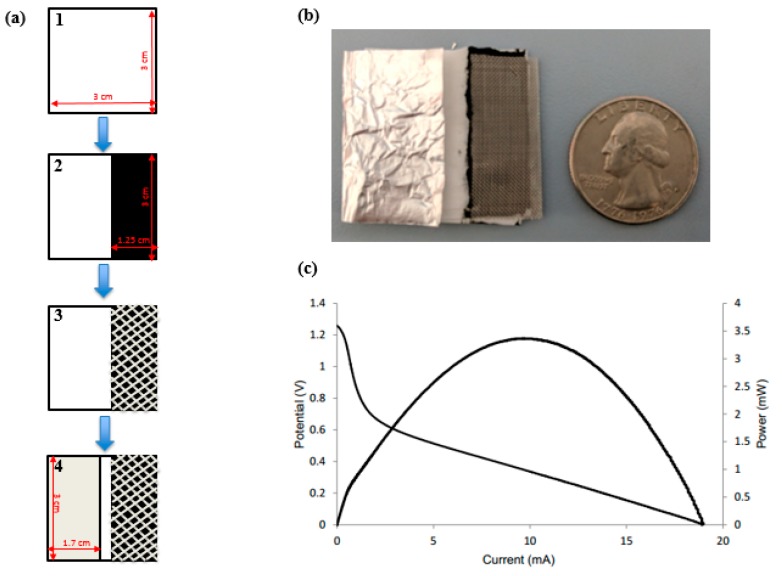
(**a**) Fabrication process of the battery in four steps: (1) folded Kim Wipe; (2) addition of activated carbon ink; (3) addition of steel mesh current collector; (4) addition of aluminum anode. (**b**) Design of a paper-based battery with the optimal dimensions of chip size and electrode size compared to the size of a US quarter. (**c**) Polarization curve of the optimal battery using 1.5 M KOH.

Figure 1a shows the fabrication process of the battery constructed from a Kim Wipe, aluminum, carbon, and steel mesh.

The electrolyte travels through the device via capillary action, requiring no external pumping. At the anode, aluminum is electrochemically oxidized to aluminum hydroxide [6]:

Al + 3OH^−^ → Al(OH)_3_ + 3e^−^, E_0_anode = −2.31 V (1)

At the cathode, oxygen is reduced to hydroxide ions:

O_2_ + 2H_2_O + 4e^−^ → 4OH^−^, E_0_cathode = 0.40 V (2)

Although the overall reduction half reaction is that described in Equation (2), the actual reaction takes place in two steps as a result of oxygen reduction on an activated carbon catalyst [19,20,21]. The four electron transfer reaction occurs on precious metals, the most common of which is platinum. However, since platinum is expensive, alternative catalysts are used including non-noble metal catalysts. The steps for the two-electron transfer on activated carbon in alkaline media are the following [22]:

O_2_ + H_2_O + 2e^−^ → HO_2_^−^ + OH^−^, E_0_ = −0.065 V (3)

HO_2_^−^ + H_2_O + 2e^−^ → 3OH^−^, E_0_ = 0.867 V (4)

The entire battery was exposed to air, allowing for oxygen to be reduced at the cathode. The assembled batteries were positioned vertically and lowered into the electrolyte, which flowed through the device.

Another equation that should be noted is the parasitic water reduction, where areas of the aluminum electrode act as both cathodic (Equation (5)) and anodic (Equation (1)) sites, creating an internal short-circuit at the anode [23]:

2H_2_O + 2e^−^ → 2OH^−^ + H_2_, E_0_ = −0.83 V (5)

This is the main cause of the difference between the thermodynamic and observed potentials in alkaline conditions [24,25,26]. When the current flows, there is also deviation from the OCV because of the electrical work performed by the cell. This deviation is the overpotential and is due to activation, ohmic, and mass transport losses. Activation losses are a result of slow reaction kinetics, while ohmic losses are a result of the resistance of the cell components, and mass transport losses arise from the depletion of reactants at the electrodes.

Moreover, heat is also liberated from the system as the anode material gets corroded [27]. In our batteries, however, heat evolution was not an issue due to the fact that we were using a relatively low KOH concentration (1.5 M) compared to the maximum KOH concentration that we tested (2.5 M).

### 3.2. Cathode/Current Collector and Electrolyte

Initial tests involved determining the appropriate cathode electrode to be tested with the aluminum anode as well as two different electrolytes, NaOH and KOH, which are most commonly used with this system [6,24]. The cathode was tested using six different electrode/current collector combinations: silver epoxy with steel mesh and activated carbon, copper with activated carbon, activated carbon with steel mesh, activated carbon alone, and silver epoxy with steel mesh. Copper [14,28], silver, and activated carbon were previously used as air cathodes in aluminum-air batteries and were thus used in our experiments. Morris et al. also mentioned that the air electrode in metal-air batteries is typically comprised of a carbon or silver catalyst [29]. With respect to amperage and standard deviation, the cathode electrode with the optimal results was composed of activated carbon with KOH electrolyte (Table 1).

Table 1 also demonstrates the fairly low power output produced from silver epoxy compared to activated carbon. This could be due to the fact that we used a silver epoxy paste, which is more commonly used as a conductive adhesive, and likely did not exhibit the same catalytic activity in oxygen reduction as pure silver. Moreover, the carbon ink was more porous compared to the silver epoxy, which exposes the electrolyte even further to air. The higher power output with activated carbon as the cathode material can be attributed to its high catalytic activity of oxygen reduction [30]. However, it was seen that activated carbon alone was more prone to tearing as the batteries became fully saturated with electrolyte. Thus, the optimized cathode electrode was instead chosen to be activated carbon with steel mesh as the current collector in KOH electrolyte, generating 2.67 mA. Although activated carbon with steel mesh in NaOH electrolyte produced slightly better results, KOH was chosen as the optimal electrolyte since NaOH showed more degradation of the aluminum during subsequent tests. The optimal cathode selection and electrolyte is shown in bold in Table 1. Copper also showed promising results (Table 1), but it showed large irreproducibility (19.4%) in NaOH and smaller amperage in KOH compared to carbon.

### 3.3. Dimensional Optimization

Four sizes were tested for the overall battery size: 1 cm^2^, 2.25 cm^2^, 4 cm^2^, and 9 cm^2^. The electrode sizes for both anode and cathode were kept constant at 0.5 cm^2^. The electrode areas for the 1 cm^2^ device were decreased to 0.48 cm^2^ to prevent the battery from being shorted. Each battery was tested with 1 M KOH. Table 2 indicates that the smallest battery size showed the greatest power due to the smaller ratio of overall battery size to electrode size.

This can be attributed to the decreased distance of the electrodes in the smaller platforms compared to the larger ones, facilitating ion transport between the electrodes. However, since the different sizes showed little variation in performance, the optimal area of the device was determined to be 2.25 cm^2^ based on its low RSD value and, hence, its reproducibility (Table 2). Another size test was conducted to determine the performance of the batteries with respect to their corresponding ratios of electrode size (Table 3).

The highest performing battery was 9 cm^2^, with a 2 cm^2^ electrode area for the anode and cathode. This size also had the highest amperage and lowest RSD. As the trend in Table 3 indicates, we attribute the increase in amperage to an increase in the electrode area (scaled with respect to platform size) for anode and cathode, allowing for further oxidation and reduction of the aluminum and oxygen at the anode and cathode, respectively. A further increase in the device size along with an increase in electrode area would lead to better performance; however, in order to maintain the consumption of electrolyte at a microfluidic scale, 9 cm^2^ was the largest size tested. Thus, subsequent experiments were carried out using a constant 9 cm^2^ device.

Once the optimal device size was determined, the anode electrode size was first varied, keeping the cathode electrode size constant at 2 cm^2^. The sizes of the anode are shown in Table 4. The highest performing battery had an anode electrode area of 6 cm^2^ and was also the size of the optimal anode electrode.

The aluminum electrode was chosen to have a larger size compared to the cathode electrode to compensate for the corrosion of the anode, therefore increasing the lifetime of the battery. This also explains the trend seen in Table 4, in which larger aluminum surface area leads to greater power. Although the 3 cm × 2 cm anode size had the largest RSD, it also had a significant jump in amperage compared to the sizes with lower RSDs. Given the goal of powering small devices, a higher amperage was important in addition to a reasonable RSD. The cathode electrode size was then varied, keeping the anode electrode size constant at 2 cm^2^ (Table 5).

The sizes of the cathode electrode are analogous to the anode size variations shown in Table 4. The highest performing battery also had a cathode electrode area of 6 cm^2^, similar to the trend seen in Table 4 due to the increased catalytic activity of reducing oxygen; however, due to its irreproducibility, the optimal cathode electrode size was 3.75 cm^2^, which had a lower relative standard deviation (5.4%). Various other combinations of cathode and anode size could have been tested as well; however, we chose to limit the number of experiments by using 2 cm^2^ as a constant for one electrode while varying the size of the other.

### 3.4. Electrolyte Optimization

With optimal dimensions of the battery, anode, and cathode determined (Figure 1b), the electrolyte concentration was varied. The device had an area of 9 cm^2^, and anode and cathode had an area of 5.1 cm^2^ and 3.75 cm^2^, respectively. The anode area was slightly decreased since the width of the electrode was previously in contact with the cathode electrode. The electrolyte concentrations varied from 0.5 M–2.5 M. During previous preliminary tests, higher concentrations of electrolyte were also tested, but were not used in this optimization due to the rapid degradation of the electrodes. The highest performing battery had a concentration of 1.5 M KOH due to higher ionic activity compared to lower concentrations (Table 6).

Although higher concentrations (2 M and 2.5 M) should have provided more current and power, the increased corrosion rate due to hydrogen evolution and heat decreases the performance of the battery. Moreover, 1.5 M KOH had a corrosion rate of aluminum that was slow enough to reuse the device over several days compared to higher concentrations of electrolyte [31]. Thus, 1.5 M KOH was the optimal concentration for the optimized dimensions of the battery. Figure 1c shows the polarization curve for a battery with optimal battery dimensions and optimal electrolyte concentration.

While the optimized device yielded a maximum current of 17.4 mA, the average OCV was 1.27 V, deviating from the theoretical voltage of 2.71 V. With the presence of more water, the competing reaction of hydrogen formation from the reduction of water is more prevalent [20], resulting in a potential mixing and decreasing the favorability of Equation (1). This is also shown in Equation (5). Chen et al. further elucidated the deviations of the anode open-circuit potential between experimentally observed values and thermodynamic predictions, attributing lower experimental values to an electronic factor, which is the asymmetry between the electrochemical steps as the bonds to the surface increase in strength with the number of adsorbates.

### 3.5. Application

**Figure 2:**
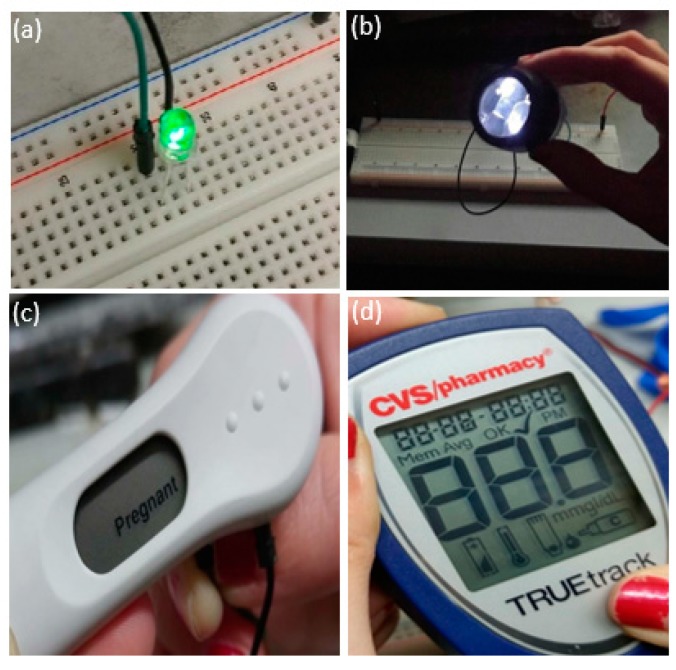
Three batteries connected in a series configuration were able to power (**a**) a green light-emitting diode (LED), (**b**) a flashlight, (**c**) a pregnancy test, and (**d**) a glucometer, all of which required a minimum of 3 V to operate.

Many small devices such as light-emitting diodes (LEDs) and diagnostic devices require a minimum of 1.5–3 V to be powered. Therefore, three batteries can be connected in a series configuration to provide sufficient voltage to power these devices. The batteries were connected in a horizontal series configuration. The anode of the first battery was connected to the device to be powered, and its cathode was connected to the anode of the second battery by a steel wire. The cathode of the second battery was connected to the anode of the third battery by a steel wire as well. The cathode of the third battery was connected to the device to be powered. The batteries could also be stacked if needed, with the proper insulation of some of the electrodes to prevent short-circuits. As expected, the voltage was additive among the three batteries and initially yielded 4 V for a triple battery configuration. This configuration generated enough power to operate LEDs over a wide range of wavelengths, a flashlight, as well as a CVS Pharmacy brand pregnancy test and glucometer (Figure 2).

### 3.6. Exhaustion Test

**Figure 3:**
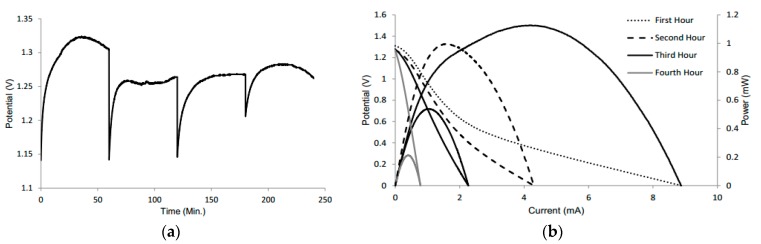
(**a**) Open-circuit voltage plot of the battery tested every hour for four hours with no addition of electrolyte after the initial 15 min. (**b**) Polarization curves of the same battery at every hour.

An exhaustion test was conducted to determine the lifetime of the battery. The battery was tested initially for 15 min with the appropriate amount of electrolyte, followed by an OCV test to observe the change in voltage without the addition of electrolyte. After four hours, there was only a slight change in the voltage, while the battery remained slightly wet (Figure 3a). The drop in voltage for each hour is attributed to a VI test, to see the change in current (Figure 3b).

The drop is expected since the VI test demonstrates the maximum amperage of the battery. Thus, it takes a few seconds for the voltage to return to its original value. After two weeks when the battery was completely dry, the voltage was 0.8 V; however, the amperage was not sufficient to power small devices. The maintained voltage can be attributed to the fact that the electrodes had only been partially degraded and that there was likely residual KOH still in the system [29]. However, the minute amount of KOH in the battery also explains the large drop in amperage. Also, because of the degradation of aluminum over time, large mass transport losses also contribute to lower amperage.

**Figure 4:**
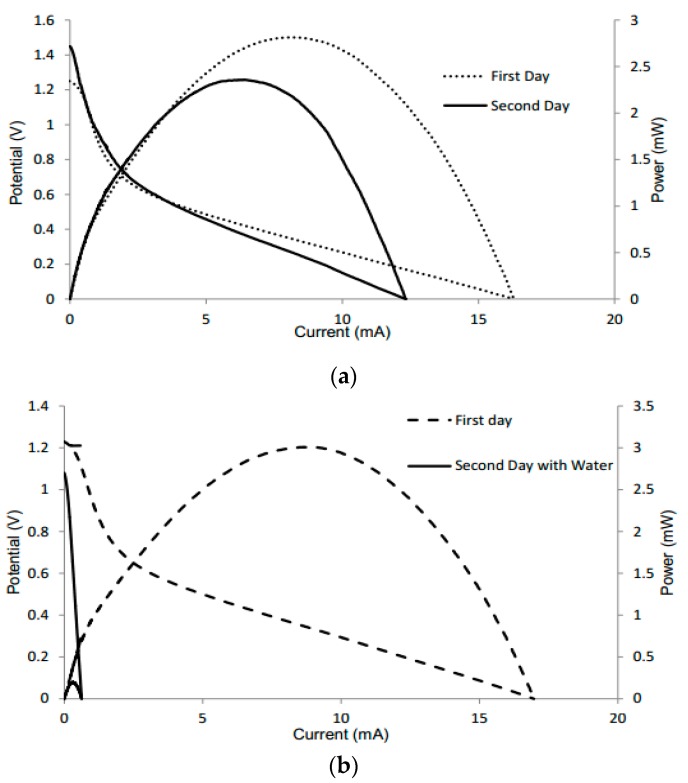
Polarization curves of different batteries tested the first day with KOH and the following day with (**a**) electrolyte and (**b**) water.

Additional tests were conducted to determine the performance of the batteries when dried and reactivated the following day with KOH or water, respectively. For the test of KOH, the device had a voltage of 1.25 V and current of 16.3 mA the first day. When KOH was added again the following day, the battery still maintained its performance, with a slight increase in voltage (Figure 4a). This increase can be attributed to the alkaline electrolyte slightly removing the oxide layer from the aluminum. However, the amperage may be lower due to corrosion of the anode. During the reaction, the electrode can be changed chemically, with the quantity of electricity that can pass being limited by the amount of metal present [32]. When retesting with water, the device had a voltage of 1.23 V and current of 16.97 mA the first day. When water was added the following day, the battery had maintained its voltage, but displayed a significant decrease in amperage (Figure 4b). This can be attributed to the water diluting the electrolyte, thus having a negative effect on the battery performance as well as increasing potential mixing, as shown in Equation (5).

## 4. Conclusions

We have described the development of an improved and inexpensive paper-based aluminum-air battery by optimizing the parameters which include the choice of the cathode electrode, electrolyte, the size of the device and electrode, and the concentration of the electrolyte. A maximum current of 17.4 mA and power of 3.0 mW was achieved with a battery that was 9 cm^2^ in size, with anode and cathode area of 5.1 cm^2^ and 3.75 cm^2^, respectively, tested with 1.5 M KOH. In a series configuration using three batteries, a maximum voltage of 4 V was obtained and was used to power a number of devices including LEDs, a flashlight, a glucometer, and a pregnancy test. These batteries provide a means of integrating environmentally friendly sources of power with small POC devices. Although there is still work being done on making the batteries more compact and user-friendly in the laboratory, the battery described in this work serves as a prototype for future modifications.

## Figures and Tables

**Table 1:** Voltage, current, and power of current collectors using a constant chip size 2.25 cm^2^ (1.5 × 1.5 cm^2^) and constant electrode size 0.5 cm^2^ (1 × 0.5 cm^2^). The optimal cathode electrode was activated carbon with stainless steel mesh. The optimal electrolyte was KOH.

1 M NaOH	**Anode**	**Cathode**	**Average Voltage**	**Average Current**	**Average Power**	**RSD of Current**
	Aluminum	Silver epoxy with steel mesh and carbon	1.38 V	0.83 mA	0.35 mW	14.8%
	Aluminum	Carbon with copper	1.21 V	2.58 mA	0.83 mW	19.7%
	Aluminum	Carbon with steel mesh	1.27 V	2.92 mA	0.55 mW	3.7%
	Aluminum	Carbon	1.28 V	2.95 mA	0.57 mW	5.3%
	Aluminum	Silver epoxy and steel mesh	1.39 V	1.36 mA	0.27 mW	23.9%
1 M KOH	**Anode**	**Cathode**	**Average Voltage**	**Average Current**	**Average Power**	**RSD of Current**
	Aluminum	Silver epoxy with steel mesh and Carbon	1.65 V	1.43 mA	0.54 mW	28.6%
	Aluminum	Carbon with copper	1.23 V	2.23 mA	0.78 mW	3.8%
	**Aluminum**	**Carbon with steel mesh**	**1.32 V**	**2.67 mA**	**0.53 mW**	**3.5%**
	Aluminum	Carbon	1.23 V	3.26 mA	0.65 mW	4.1%
	Aluminum	Silver epoxy and steel mesh	1.43 V	2.13 mA	0.46 mW	54.4%

**Table 2:** Voltage, current, and power of varying chip sizes using constant electrolyte concentration (1 M KOH) and constant electrode size 0.5 cm^2^ (1 cm × 0.5 cm). The optimal chip size was 2.25 cm^2^ (1.5 cm × 1.5 cm).

Platform Size	Average Voltage	Average Current	Average Power	RSD of Current
1 cm × 1 cm	1.30 V	2.69 mA	0.54 mW	10.0%
**1.5 cm × 1.5 cm**	**1.32 V**	**2.67 mA**	**0.53 mW**	**3.5%**
2 cm × 2 cm	1.33 V	2.32 mA	0.49 mW	7.1%
3 cm × 3 cm	1.33 V	2.70 mA	0.50 mW	7.3%

**Table 3:** Voltage, current, and power of varying chip sizes with proper electrode sizes using ratios with constant electrolyte concentration (1 M KOH). The optimal chip was 9 cm^2^ (3 cm × 3 cm) with their respective ratios of catalysts.

Platform Size	Electrode Size	Average Voltage	Average Current	Average Power	RSD of Current
1 cm × 1 cm	0.67 cm × 0.33 cm	1.27 V	2.68 mA	0.50 mW	15.2%
1.5 cm × 1.5 cm	1.0 cm × 0.5 cm	1.32 V	2.67 mA	0.53 mW	3.5%
2 cm × 2 cm	1.3 cm × 0.67 cm	1.32 V	3.07 mA	0.67 mW	4.8%
**3 cm × 3 cm**	**2 cm × 1 cm**	**1.30 V**	**6.00 mA**	**1.12 mW**	**3.4%**

**Table 4:** Voltage, current, and power of a 9 cm^2^ (3 cm × 3 cm) chip with varying aluminum area and 2 cm^2^ (2 cm × 1 cm) cathode area with constant 1 M KOH. The optimal area size for aluminum was 6 cm^2^ (3 cm × 2 cm).

Anode Size	Average Voltage	Average Current	Average Power	RSD of Current
1.5 cm × 0.5 cm	1.33 V	3.22 mA	0.62 mW	6.0%
2 cm × 1 cm	1.30 V	6.00 mA	1.12 mW	3.4%
2.5 cm × 1.5 cm	1.33 V	8.85 mA	1.62 mW	10.0%
**3 cm × 2 cm**	**1.34 V**	**11.10 mA**	**2.07 mW**	**11.7%**

**Table 5:** Voltage, current, and power of a 9 cm^2^ (3 cm × 3 cm) chip with varying cathode area and 2 cm^2^ (2 cm × 1 cm) aluminum area with constant 1 M KOH. The optimal area size for the cathode was 3.75 cm^2^ (2.5 cm × 1.5 cm).

Cathode Size	Average Voltage	Average Current	Average Power	RSD of Current
1.5 cm × 0.5 cm	1.34 V	2.56 mA	0.54 mW	5.1%
2 cm × 1 cm	1.30 V	6.00 mA	1.12 mW	3.4%
**2.5 cm × 1.5 cm**	**1.33 V**	**10.41 mA**	**1.97 mW**	**5.4%**
3 cm × 2 cm	1.38 V	11.08 mA	2.16 mW	20.2%

**Table 6:** Voltage, current, and power of a 9 cm^2^ (3 cm × 3 cm) chip with aluminum area of 5.1 cm^2^ (3 cm × 1.7 cm) and carbon area of 3.75 cm^2^ (3 cm × 1.25 cm) with varying KOH concentrations. The optimal concentration was 1.5 M KOH.

KOH Concentration	Average Voltage	Average Current	Average Power	RSD of Current
0.5 M	1.35 V	10.12 mA	1.96 mW	12.1%
1 M	1.31 V	14.04 mA	1.81 mW	1.8%
**1.5 M**	**1.27 V**	**17.43 mA**	**3.05 mW**	**8.6%**
2 M	1.24 V	16.81 mA	2.80 mW	5.2%
2.5 M	1.24 V	16.86 mA	2.85 mW	12.4
